# Identification of Differentially Expressed Genes in Human Colorectal Cancer Using RNASeq Data Validated on the Molecular Level with Real-Time PCR

**DOI:** 10.1007/s10528-023-10593-5

**Published:** 2023-12-14

**Authors:** Aya M. A. Elsayed, Mariam Oweda, Asmaa M. Abushady, Maha Alhelf, Shaimaa R. M. Khalil, Mohamed S. Tawfik, Walid Al-Atabany, Mohamed El-Hadidi

**Affiliations:** 1 School of Biotechnology, Nile University, Giza, Egypt; 2 School of Information Technology and Computer Science, Nile University, Giza, Egypt; 3 Department of Agricultural, Forest and Food Sciences, University of Torino, Turin, Italy; 4 Bioinformatics Group, Center for Informatics Sciences (CIS), School of Information Technology and Computer Science (ITCS), Nile University, Giza, Egypt; 5 Genetic Department, Faculty of Agriculture, Ain Shams University, Cairo, Egypt; 6 Medical Biochemistry and Molecular Biology Department, Faculty of Medicine, Cairo University, Cairo, Egypt; 7 Oil Crops Biotechnology Lab, Agricultural Genetic Engineering Research Institute (AGERI), Agricultural Research Center (ARC), 9 Gamaa Street, Giza, 12619 Egypt; 8 Institute of Cancer and Genomic Sciences, College of Medical and Dental Sciences, University of Birmingham Dubai Campus, Dubai, United Arab Emirates

**Keywords:** Colorectal cancer, GEO, DEGs, GProfiler, RNA-Seq, DESeq2 packages, *Withania somenifra*, MMP7, TCF21, VEGFD

## Abstract

Colorectal cancer (CRC) is a prevalent cancer with high morbidity and mortality rates worldwide. Late diagnosis is a significant contributor to low survival rates in a minority of cases. The study aimed to perform a robust pipeline using integrated bioinformatics tools that will enable us to identify potential diagnostic and prognostic biomarkers for early detection of CRC by exploring differentially expressed genes (DEGs). In addition to, testing the capability of replacing chemotherapy with plant extract in CRC treatment by validating it using real-time PCR. RNA-seq data from cancerous and adjacent normal tissues were pre-processed and analyzed using various tools such as FastQC, Kallisto, DESeq@ R package, g:Profiler, GNEMANIA-CytoScape and CytoHubba, resulting in the identification of 1641 DEGs enriched in various signaling routes. MMP7, TCF21, and VEGFD were found to be promising diagnostic biomarkers for CRC. An in vitro experiment was conducted to examine the potential anticancer properties of 5-fluorouracile, *Withania somnifera* extract, and their combination. The extract was found to exhibit a positive trend in gene expression and potential therapeutic value by targeting the three genes; however, further trials are required to regulate the methylation promoter. Molecular docking tests supported the findings by revealing a stable ligand-receptor complex. In conclusion, the study’s analysis workflow is precise and robust in identifying DEGs in CRC that may serve as biomarkers for diagnosis and treatment. Additionally, the identified DEGs can be used in future research with larger sample sizes to analyze CRC survival.

**Supplementary Information:**

The online version contains supplementary material available at 10.1007/s10528-023-10593-5.

## Introduction

Colorectal cancer (CRC) is a prominent global health concern, it is considered the third most commonly diagnosed cancer and the second leading cause of cancer-related mortality worldwide, as reported by the International Agency for Research on Cancer (IARC). The incidence of CRC is raising in many countries, largely attributable to lifestyle and dietary changes. According to the IARC’s 2020 estimates, there were approximately 1.9 million new cases and 935,000 deaths worldwide due to CRC. CRC is often asymptomatic in its early stages, making it a challenging disease to diagnose. However, most CRCs develop from precancerous polyps, which can be detected and removed through screening. The stage of CRC at diagnosis is a major prognostic factor, with patients with localized disease having significantly better survival outcomes than those with regional or distant metastasis. Individuals who are diagnosed with early stage malignancies classified as Dukes A (T^1/2^N^0^M^0^) exhibit a 5-year survival rate of 93.2%. In contrast, individuals diagnosed with advanced-stage cancers categorized as Dukes C (T^3/4^N^1/2^M^0^) experience a significant decrease in the 5-year survival rate, which lowers to 47.7% (Morris et al. 2010). Hence, early recognition of precancerous lesions in the colorectal significantly contributes to enhancing the 5-year rate of survival (Shah et al. 2014). Besides, the improvement of the prognosis as well the increase of survival rates among patients are affected by CRC on timely treatment and early detection (Arhin et al. 2022; Khazaei et al. 2018).

It is difficult to recognize tumors smaller than 1 cm using traditional diagnostic techniques because the sensitivity and specificity of conventional tumor markers are inadequate. Which led to the necessitating identification of corresponding biomarkers (Mármol et al. 2017; Li et al. 2018).

The chemotherapeutic agent known as 5 fluorouracil (5-FU) is extensively utilized in the management of various malignancies, such as colorectal and breast cancers, as well as aerodigestive tract tumors (Sargent et al. 2001). *Withania somnifera* (L.) Dunal is the scientific name of a plant commonly known as ashwagandha. In Ayurvedic researches, it is sorted as a rejuvenator and it is utilized to enhance mental and physical states, restore the body when incapacitated, and promote longevity. *W. somnifera* has been found to be beneficial for various neurological conditions, including Alzheimer’s disease, epilepsy, cerebral ischemia, Parkinson’s disorders, and tardive dystonia, as reported by Kulkarni and Dhir (2008); Mukherjee et al. (2021). In addition to its neurological benefits, *W. somnifera* exhibits a number of other pharmacological qualities, including anti-inflammatory, anti-diabetic, cardioprotective, and anti-tumor capabilities (Behl et al. 2020; Logie and Vanden Berghe 2020).

Bioinformatics analysis has a great role in differential expression analysis to identify DEGs in CRC and other cancer types. Several investigations have been carried out to examine gene expression in CRC through the utilization of microarray and RNA-sequencing technology, alongside publicly available databases such as the Gene Expression Omnibus (GEO) and The Cancer Genome Atlas (TCGA). Guo et al. (2017) conducted a study with the objective of identifying potential genes and pathways in CRC by utilizing four GEO cohorts where a total of 292 differentially expressed genes (DEGs) were identified from the four datasets, with 165 genes upregulated in addition to 127 genes downregulated. In their study, Wu et al. (2017) conducted an analysis of gene and microRNA expression profiles obtained from GEO datasets. Their findings revealed the identification of seven downregulated differentially expressed miRNAs and 13 upregulated DE miRNAs, as well as approximately 600 upregulated DEGs as well as 283 downregulated genes (Liang et al. 2016; Sun et al. 2019).

Additionally, molecular docking plays a role in validating the anti-tumor effect of *W. somnifera* extract against different targets, in our case the resulted DEGs, by finding the ligand-receptor interaction that has greater stability by lower binding affinity (Tantawy et al. 2020).

Based on the aforementioned literature, the objective of this study is to identify the most notable and robust DEGs associated with CRC through the utilization of integrated bioinformatics methodologies and test the cytotoxicity effect of 5-FU (as a chemotherapy treatment used for CRC) and *W. somnifera* extract on HCT-116 cells. In addition, we aim to test the expression of the most three potential DEGS resulted from in-silico analysis by q-PCR to validate them on a molecular level. Finally, we intent to validate the efficacy of *W. somnifera* extract by knowing its binding affinity to the target genes compared to other ligands using AutoDock tools. Thus, this study will give more details about the ability of our robust bio-informatics pipeline to investigate the most potential differential gene expression in CRC, which may contribute in recognizing new CRC bio-markers, as well as the capability to replace the chemotherapy as 5-FU with *W. somnifera* extract.

## Methodology

### Data Retrieval

An investigation for the availability of RNA-Seq transcriptomic data related to CRC from the GEO (Barrett et al. 2012) and Sequence Read Archive (SRA) were done. These databases are managed by the National Center for Biotechnology Information for profiling gene expression and RNA methylation. Microarrays and RNA-Seq techniques provide high-throughput genomic data screening. The accession number GSE156451 was used to retrieve raw RNA sequence paired-end data for 50 samples, 25 tumor samples, and 25 normal tissue samples.

### Pre-processing of Raw Data

#### Raw Data Quality Check

The quality of the reads obtained with FastQ was checked using FASTQC (Andrews et al. 2010) followed using MultiQC (Ewels et al. 2016) for combining FASTQC reports. The adapters in the raw data were trimmed using Cutadapt (version 4.1) (Martin 2011) with normal parameters. Read quality was retested after adapter trimming which were improved using by Trimmomatic and remove contamination that appeared as over-represented sequences. Reads with a Phred quality score of > 25 and minimum length of 36 nt were selected.

#### Reads Mapping to Reference Genome and Gene Expression Quantification

Good-quality reads were mapped to the latest human genome assembly (GRCh38), which was retrieved from the Ensemble genomic browser with the corresponding annotation GTF file using Kallisto pseudo-aligner (version 0.46.0) (Bray et al. 2016) for quantifying the abundance of RNA-seq data. Kallisto can process both single- and paired-end reads and provides the number of transcripts per million mapped reads (TPM). In this study, the pair-ended default running mode were used, in which, FASTQ files were represented as pairs. Gene expression quantification was performed automatically through Kallisto after the pseudoalignment step and a quantification table was generated as. tsv file format, which was then used for the DeSeq2 R package.

### Differential Gene Expression Analysis

Gene expression quantification data were transferred as gene expression data to the Deseq2 package in R software (Love et al. 2014) to investigate significant DEGs in both colorectal tumor samples and nearby non- cancerous samples (control). Relevant parameters ((*|log*2*FC|*> 1*.*2 and *p*adj < 0*.*05) were set to filter out the DEGs. The DESeq2 package were used to pair the information for all samples by employing a design of the form “Samples + Tissue” for the Sample Table. The “samples” column was used to record the patient’s identification number, while the “tissue” column was used to designate the tissue type, whether normal or cancerous tissue.

#### Visualization of Differentially Expressed Genes

The data were visualized by RStudio (R version 4.2.0) to obtain the maximum perspective on the resulting data, diverse types of plotting were implemented and maintained. Among these plots is the volcano plot, which is a scatter plot that represents the differential expression of genes in this study. In addition, a heatmap was applied using a heatmap function to plot the value versus frequency, in which annotation colors were established, and data scaling was performed to provide a color scale for each map’s values

### Network Construction

The GeneMANIA-Cytoscape app was used to study the interactions and correlations between DEGs in the dataset, in which direct physical interactions, pathway interactions, and co-expression interactions were only considered for the *Homo sapiens* database. The top ten most closely related genes were included in the network construction. The STRING database was used to visualize gene interactions based on the K-means clustering method, enabling the identification of significant hub gene nodes for each network. Moreover, the CytoHubaa blugin in Cytoscape was used to calculate the Maximal Clique Centrality and other 6-centralities as: closeness, bottleneck, betweenness, eccentricity, radiation, and stress (Chin et al. 2014).

### Functional Enrichment Analysis

The g:Profiler enrichment analysis was carried out by analyzing the KEGG and Reactome pathways to explore related pathways, biological processes, and molecular functions (MFs) of the aforementioned DEGs. The g:Profiler cut-off score was used with a detection rate threshold of 0.05 FDR. The parameters utilized to detect significant genes included the number of genes in the dataset (1641), the statistical domain was limited to annotated genes, and a significant threshold of Benjamini–Hochberg FDR was applied, along with a user threshold of 0.05. These parameters were used as the basis for the selection of highly expressed genes.

### Cell Viability Assay

#### Cell Culture

HCT-116 cells derived from CRC tissues were procured from Nawah Scientific Inc. (Mokatam, Cairo, Egypt). These cells were cultured in RPMI media supplemented with 100 mg/mL streptomycin, 100 units/mL penicillin, and 10% heat-inactivated fetal bovine serum under humidified conditions in a 5% (v/v) CO_2_ atmosphere at 37 °C.

#### Sulforhodamine B (SRB) Cytotoxicity

Cell viability was assessed using the SRB assay to ascertain the IC50 concentration for each treatment group. A suspension containing 5 × 10^3^ cells was seeded in each well of a 96-well plate and subsequently incubated in complete media for 24 h. Subsequently, the cells were treated with 100 L of media containing various drug concentrations. After 72 h of drug exposure, the cells were fixed by substituting the media with 150 L of 10% trichloroacetic acid (TCA) and incubated at 4 °C for 1 h. The TCA solution was removed, and the cells were washed five times with distilled water.

An amount of 70 L SRB solution containing 0.4% (weight/volume) was introduced and incubated under light-deprived conditions at ambient temperature for 10 min. The plates were washed three times with 1% acetic acid solution, followed by an overnight period of air-drying. Subsequently, 150 L of TRIS (10 mM) was added to facilitate dissolution of the protein-bound SRB stain. The absorbance was then measured at 540 nm with the aid of a BMG LABTECH®- FLUOstar Omega microplate reader (Ortenberg, Germany), according to the sources cited (Skehan et al. 1990; Allam et al. 2018). The HCT-116 cell lines were categorized into four groups, as shown in Table 1, based on drug type and concentration. Each drug was administered at five different concentrations to determine the IC50 values.

**Table 1:** Classification of study groups

Group number	Culture description
Group I	Untreated cancer HCT-116 cells (control) cultured for 72 h
Group II	HCT-116 cells treated with 5-FU with five different concentrations cultured for 72 h
Group III	HCT-116 cells treated with Withanolide extract cultured for 72 h
Group IV	HCT-116 cells treated with combined 5-FU and Withanolides ex tract with 1:1 ratio cultured for 72 h

### RNA Extraction and cDNA Synthesis

Total RNA was extracted and purified from the cell line pellets using the Qiagen RNeasy Mini kit (Cat No.74104) following the manufacturer’s instructions. RNA samples were quantified, and their quality was assessed using a Nanodrop spectrophotometer at A230, A260, and A280. First-strand cDNA synthesis was performed on RNA samples obtained from the four groups using the RevertAid First-Strand cDNA Synthesis Kit (Cat K1622, Thermo Scientific^TM^) according to the manufacturer’s instructions. The cDNA was synthesized using a Bio-Rad TM 100 Thermal Cycler.

### Real-Time PCR to Test Gene Expression

The cDNA was then amplified with the TB Green Permix EXTTaq PCR Master Kit in a 48-well plate using the Stratagene Mx3005P, Agilent Technologies, as follows: 30 s. at 95 °C for enzyme activation, followed by 45 cycles of 5 s at 95 °C, 30 s at 60 °C, and 30 s at 72 °C for the amplification step. A quantity of 0.25 uM was used from both primers specific for each target gene. The total volume of the reaction was completed with ddH_2_O of 20 µL. The relative gene expression foldChange was calculated using the PCR analysis R package according to the equation $2^{ - \Lambda \Lambda C_{\text{t}} }$.

### Molecular Docking and the Prediction of Pharmacological Targets

The X-ray crystallographic structure of each gene was obtained from the RCSB PDB (Protein Data Bank) (RCSB.org) while the 2D structure of each ligand was obtained from PubChem. The Drug Gene Interaction Database (DGIdb) and Drug Interaction Checker were used to identify possible druggable gene interactions of our target genes and drug–drug interactions, respectively. The *Withania somnifera* extract was searched in the drug interaction checker to identify ligands that may have common active ingredients.

Precise docking was performed using AutoDocvina v.1.2.0. The binding energy (measured in kcal/mol) was used to assess the precision of the docking process with 70 runs taken and a maximum of 2000 interactions, as well a population size of 200, and an energy threshold of 100. Furthermore, during each iteration the minimum torsional, transitional, and rotational values were evaluated. Then, the configuration with the lowest energy was selected. The hydrophobic and electrostatic preferences were set to 1. To identify the binding site of the target, grid box dimensions were adjusted using Mgltools to ensure a distance of 1 Å. Finally, Pymol software was used to visualize ligand-target binding.

### Methodology Diagram

See Fig. 1.

**Fig. 1:**
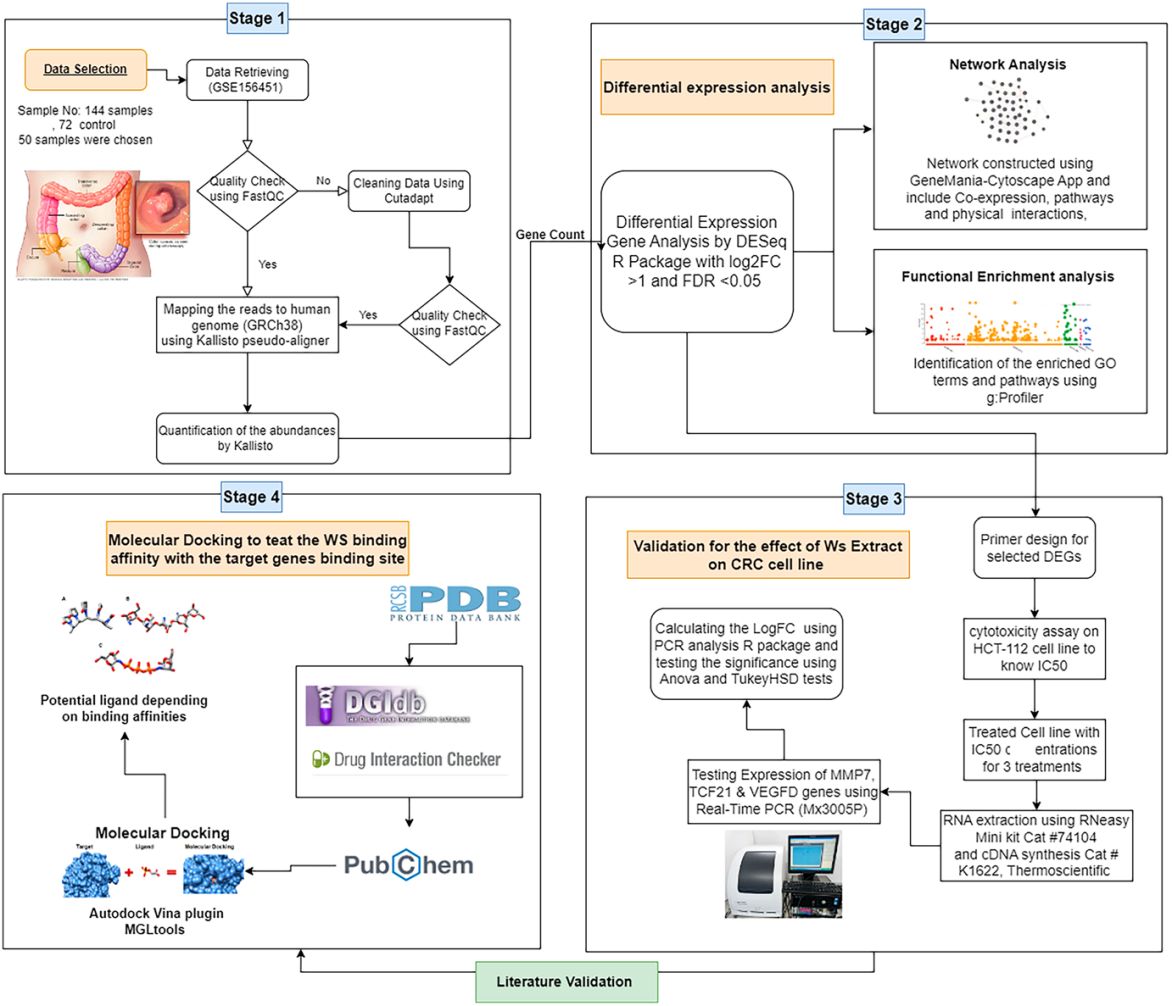
Diagrammatic methodology pipeline

## Results and Discussion

### Check for Data Quality

The sequence count in the 100 samples ranged from 10 M to 45 M, for all samples, the duplicated read counts accounted for more than 50% of the total read count, which was expected in RNA-seq experiments. As they showed some adapter contamination, we applied cutadapt software to cut this part from the read.

**Fig. 2:**
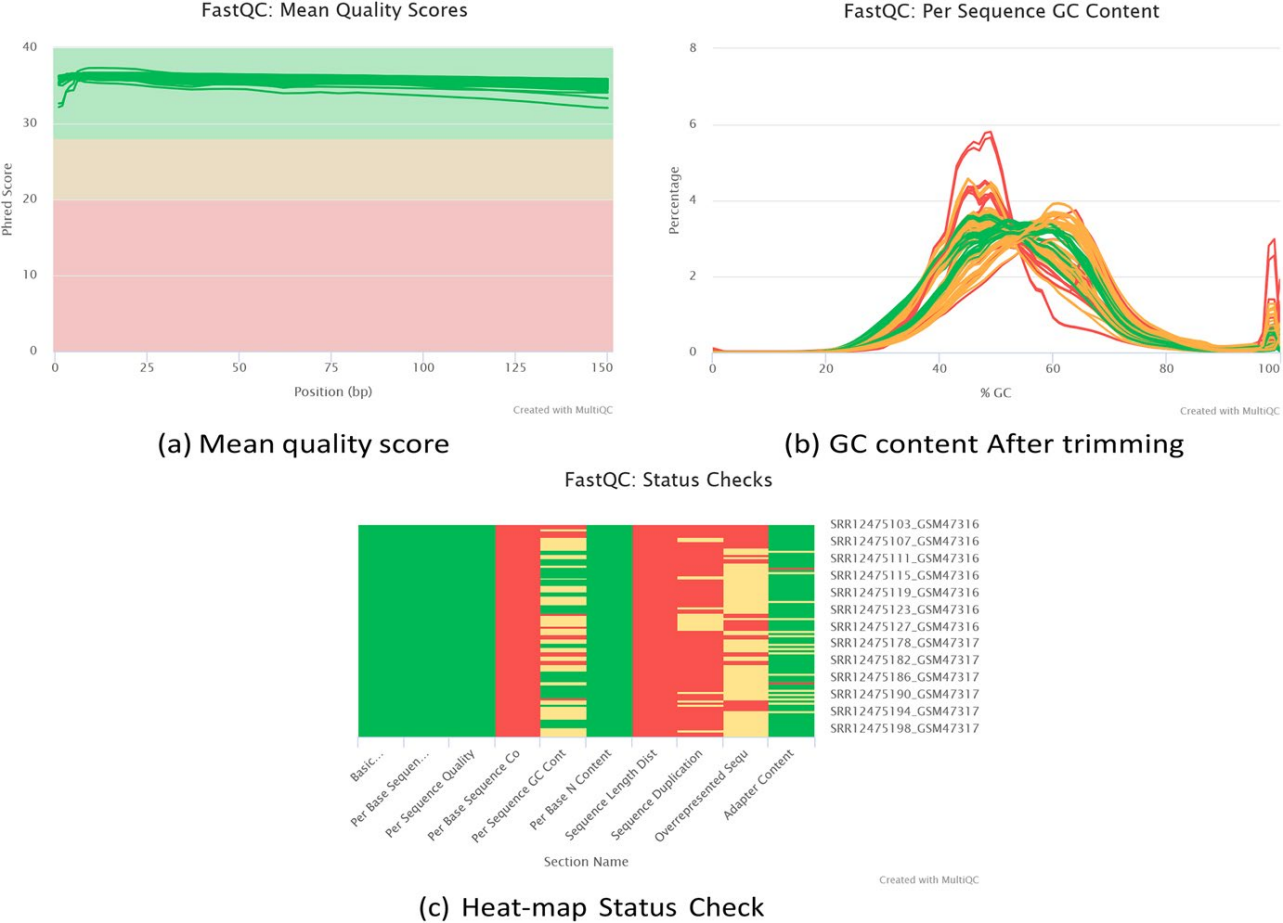
**a** Mean quality score, **b** GC content after trimming and **c** Heat-map status check

Although the quality Phred score of the samples ranged from 30 to 40, the quality of the reads in terms of adapters was not significant, as the MultiQC report showed remaining adapters in 17 samples (two of them failed and others were not satisfied). The over-represented sequences were analyzed by BLAST and were shown to be bacterial and viral contaminants in the GEO datasets. Consequently, quality trimming was applied to the trimmed reads, which resulted in the removal of approximately 85 percent of the low-quality reads or contaminants, as shown in Fig. 2a–c.

### Differentially Gene Exression

The Kallisto aligner succeeded in quantifying 204,563 reads in each sample and resulted in an expression matrix containing the ensemble transcript ID for each transcript and its abundance. Screening the control and tumor samples using DESeq2 with the previously mentioned parameters resulted in the identification of 1641 DEGs, including 773 upregulated genes and 869 downregulated genes. The top 20 upregulated and 20 downregulated genes were investigated for use in the wet lab experiment validation using real-time PCR, as shown in Tables 2 and 3. Matrix metallopeptidase 7 (MMP7) has crucial regulatory roles in numerous pathophysiological processes in humans. Since its discovery, MMP-7 has been identified as a regulatory protein in wound healing, bone development, and remodelling. Subsequently, it was shown that MMP-7 regulates the formation and development of malignancies, mediates the proliferation, differentiation, metastasis, and invasion of several types of cancer cells through multiple methods, and is overexpressed in cancer cells. (Liao et al. 2021). In addition, transcription factor-21 (TCF-21), also called epicardin, capsulin, or Pod1, is involved in epithelial-mesenchymal interactions, including epithelial differentiation and branching morphogenesis, during kidney and lung morphogenesis. Possible participant in the specification or differentiation of one or more subsets of epicardial cell types Helix-loop-helical protein structures. TCF-21, which is positioned on 6q23, is known to function as a tumor suppressor and is dysregulated in several malignancies, including breast cancer, gastric cancer, and clear cell renal cell carcinoma (Liao et al. 2021). Furthermore, the vascular endothelial growth factor D (VEGFD) gene, which is stimulated by chronic inflammation, is essential for tumor angiogenesis, tumor growth, and tumor spread (Hu et al. 2015).

**Table 2:** Top 20 up-regulated CRC DEGS generated by DEseq 2 targeted to validated on the molecular level by real-time PCR

Upregulated						
Gene name	Base mean	log2 fold change	lfcSE	Stat	*p* value	*p*adj
REG1B	201.6130025	8.210027998	0.810261866	10.13256126	3.96E−24	1.14E−21
REG1A	907.6623784	7.592851199	0.675026975	11.24821893	2.36E−29	1.74E−26
AQP5	22.97142931	6.060476648	0.949285775	6.384248883	1.72E−10	2.94E−09
CST1	68.0183565	5.825231796	0.579127798	10.05862923	8.42E−24	2.32E−21
MMP7	98.77105432	5.756760847	0.460492148	12.50132249	7.34E−36	9.74E−33
HOXC11	7.701043003	5.711354675	1.310256525	4.358959156	1.31E−05	7.86E−05
SOX14	7.070523139	5.594186265	0.829915138	6.740672637	1.58E−11	3.36E−10
KRT6B	29.99421449	5.503181412	0.667536138	8.244020207	1.67E−16	8.12E−15
TCN1	8.370082253	5.488377381	0.645123392	8.507484686	1.78E−17	1.10E−15
SLC35D3	50.13405944	5.461771198	0.559950257	9.754029279	1.77E−22	3.56E−20
BLACAT1	17.53847493	5.286971702	0.491884598	10.74839856	6.03E−27	2.63E−24
CXCL5	364.336273	5.252258228	0.60154759	8.731243077	2.52E−18	1.90E−16
IGHV1-18	3.983131453	5.243572499	1.925171643	2.723690908	0.006455689	0.018930217
EN2	7.055644805	5.199768465	0.777228321	6.690142811	2.23E−11	4.49E−10
BHLHA9	3.878381054	5.084781423	0.91360151	5.565644724	2.61E−08	2.79E−07
KRT23	34.41500991	5.063056312	0.696917048	7.264933936	3.73E−13	1.02E−11
IGHJ5	5.356621311	5.035636993	1.846490733	2.727139056	0.006388611	0.018766722
DKK4	7.251232359	4.981910386	0.775897161	6.420838531	1.36E−10	2.39E−09
HOXC6	4.183523445	4.916792142	0.962467193	5.108529595	3.25E−07	2.73E−06
DEFA6	32.46286224	4.879990389	0.844454035	5.778870354	7.52E−09	9.20E−08

**Table 3:** Top 20 down-regulated CRC DEGS generated by DEseq 2 targeted to validated on the molecular level by real-time PCR

Downregulated						
Gene name	Base mean	log2 fold change	lfcSE	stat	*p* value	*p*adj
OTOP3	61.36179242	− 6.869036153	0.539025107	− 12.7434438	3.39E−37	5.62E−34
BEST4	3844.503292	− 5.965763559	0.380857819	− 15.66401754	2.67E−55	1.77E−51
RPS15AP11	8.996398961	− 5.046657951	0.553711881	− 9.114230931	7.92E−20	8.34E−18
NGB	2.927501044	− 4.95305145	0.880471674	− 5.625452355	1.85E−08	2.07E−07
GUCA2B	3244.294719	− 4.715719781	0.511455991	− 9.220186811	2.97E−20	3.58E−18
KRT24	60.81807867	− 4.493349534	0.609855468	− 7.36789251	1.73E−13	5.11E−12
SGCG	5.987174233	− 4.438085177	0.605667848	− 7.327589189	2.34E−13	6.75E−12
SPIB	123.2911324	− 4.303535274	0.360826326	− 11.92688826	8.57E−33	8.12E−30
PRIMA1	11.5785735	− 4.162036708	0.508066854	− 8.191907571	2.57E−16	1.22E−14
DPT	348.9684246	− 4.110998269	0.463725599	− 8.865152757	7.64E−19	6.25E−17
CFD	195.631645	− 3.934852639	0.607830582	− 6.473600959	9.57E−11	1.73E−09
ANGPTL7	5.295501587	− 3.891590728	0.66193	− 5.879157503	4.12E−09	5.29E−08
NPY	4.268423235	− 3.878289437	0.513668109	− 7.550185359	4.35E−14	1.47E−12
DAO	6.587028512	− 3.836137149	0.542269952	− 7.074220387	1.50E−12	3.73E−11
VEGFD	9.067927261	− 3.749437904	0.467232447	− 8.024780662	1.02E−15	4.44E−14
FOXL3-OT1	3.131242191	− 3.672212993	0.57906559	− 6.341618393	2.27E−10	3.69E−09
UGT2B17	213.6165283	− 3.660321819	1.102846649	− 3.318976235	0.000903481	0.003374693
C14orf180	1.554249108	− 3.65426278	0.597807278	− 6.112777338	9.79E−10	1.42E−08
USH1G	3.726635769	− 3.645636179	0.507387746	− 7.185108835	6.72E−13	1.76E−11
GUCA2A	11285.20676	− 3.637593867	0.491670293	− 7.398441433	1.38E−13	4.19E−12

### Visualization of DEGs

**Fig. 3:**
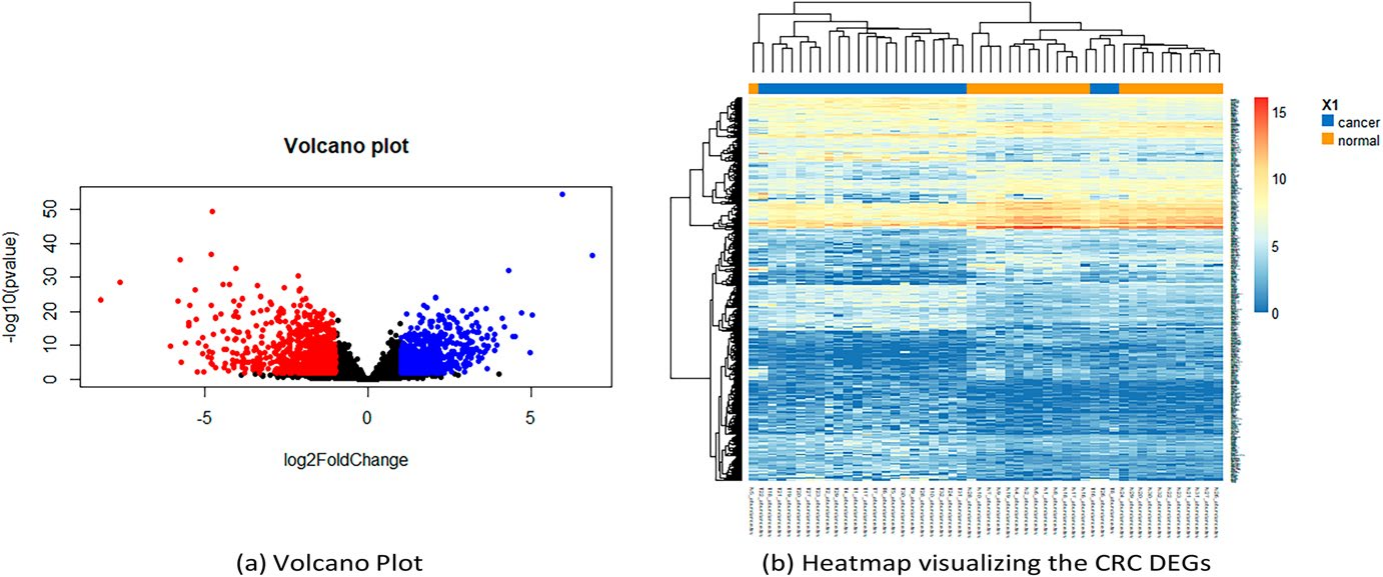
**a** Volcano plot and **b** heatmap visualizing the CRC DEGs

Typically, the most upregulated genes lie towards the right of the zero value of the x-axis (logFoldChange), while the most downregulated genes are towards the left. Additionally, the most statistically significant genes are usually scattered towards the top, as shown in Fig. 3a.

Depending on the log2FoldChange value (positive or negative), we determined which of the differentially expressed results were upregulated and which were downregulated. When comparing the number of up-regulated genes with their respective plots, it can be observed that the colored spots in blue on the right side of the volcano plot matched the aforementioned number of up-regulated genes. It can be concluded that the case would be similar to that of the down-regulated genes. Therefore, volcano plots can be a reliable visualization method for identifying significant DE genes.

Based on the RNA-Seq data, only the first 500 DE genes were selected and visualized using a heatmap to demonstrate the relationship between the values and frequencies of these genes, as shown in Fig. 3b. Heatmap clustering analysis was used to cluster the DEGs into two groups. Considering the high number of genes observed in this section. Furthermore, the top bar indicates which of them are normal (orange) patients and which are patients with primary tumors (blue).

### Network Analysis

**Fig. 4:**
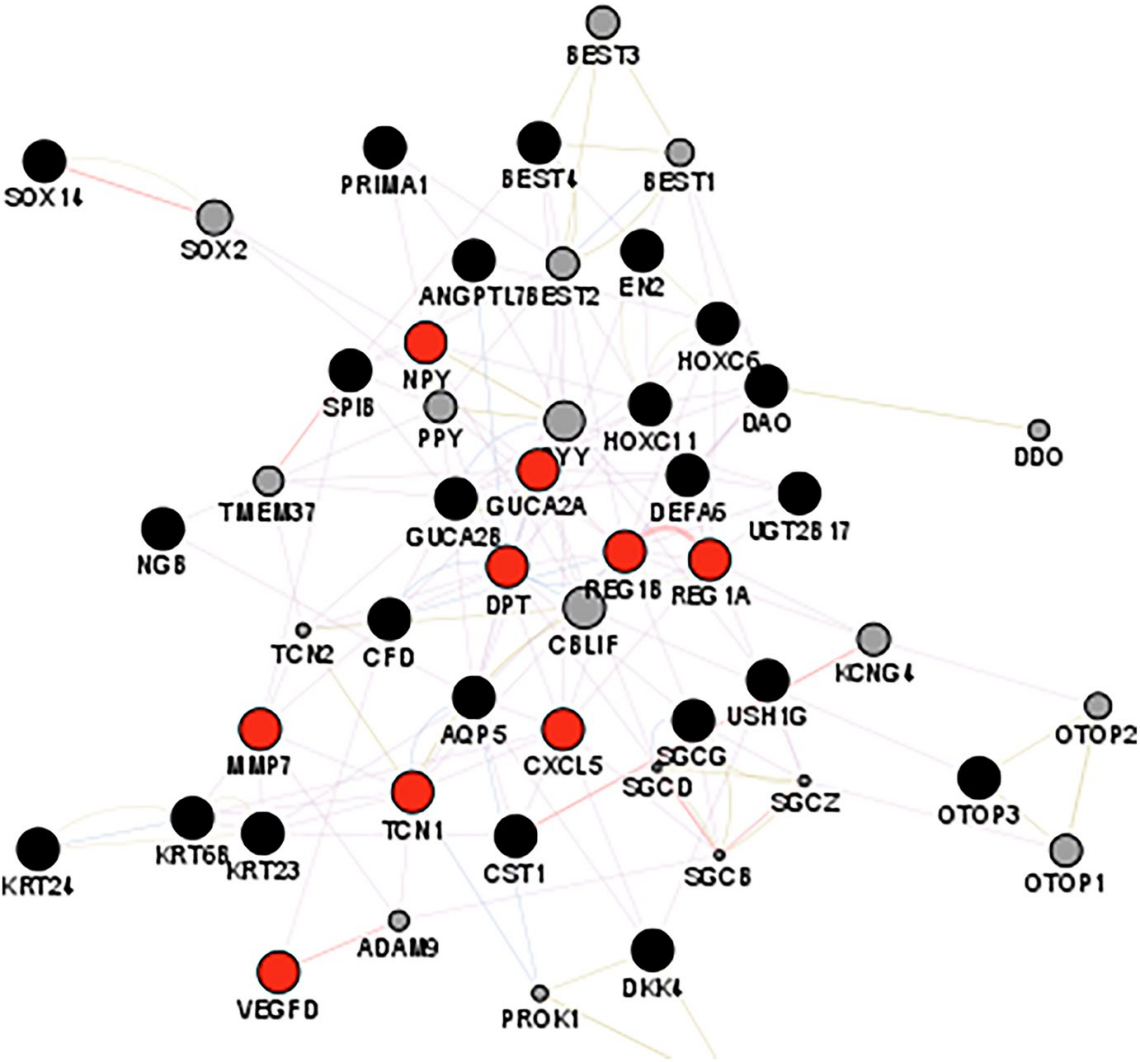
Network analysis of the top 40 CRC DEGs

**Fig. 5:**
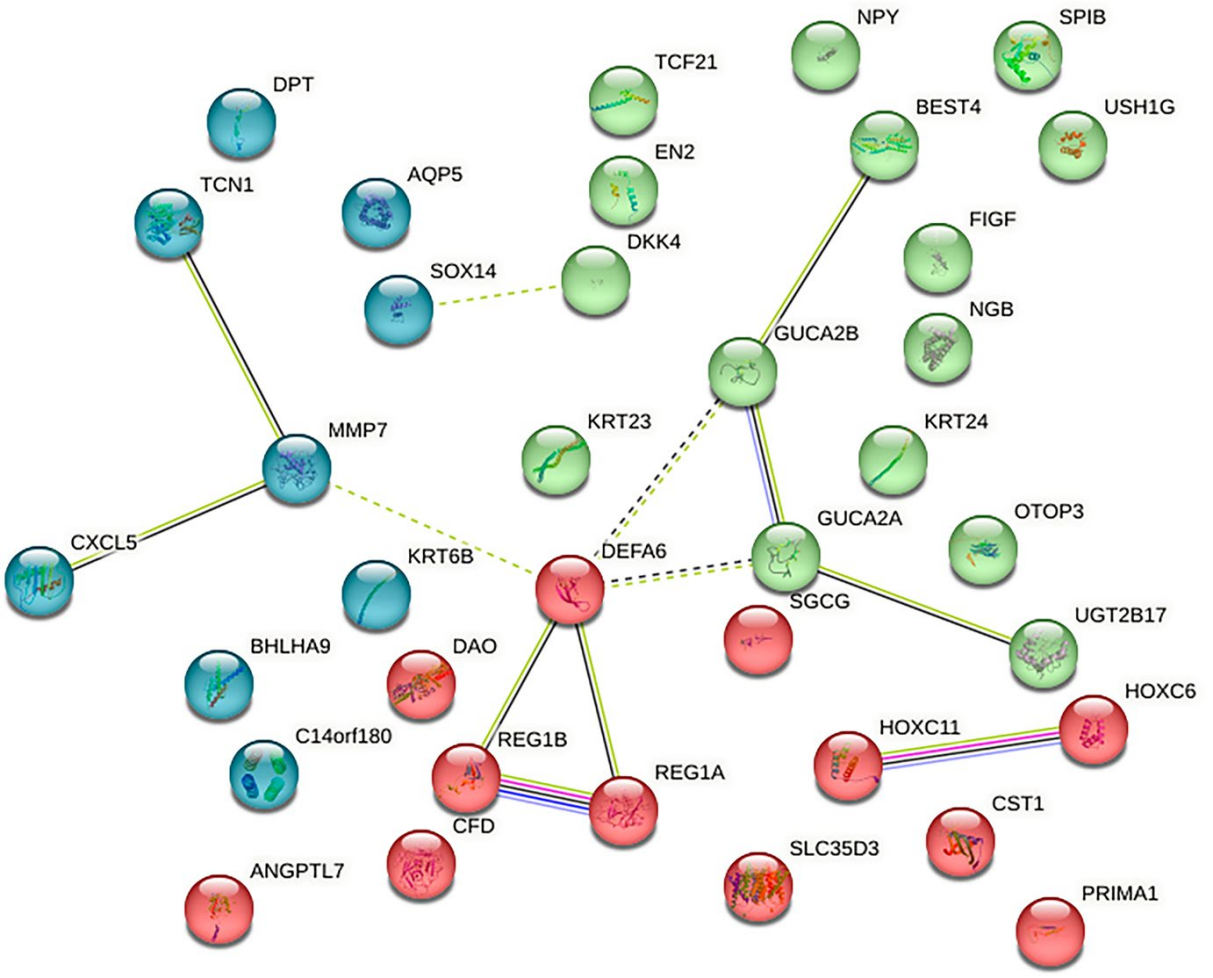
Clustering network of the top 40 CRC DEGs after applying K-means clustering method

DEGS interactions were investigated using GeneMania-cytoScape and CytoHubba to rank the nodes depending on MCC calculation and other centralities, such as closeness and betweenness. According to CytoHubba, REG1B, TCF-21, MMP7, and VEGF-D were among the top 20 ranking genes, depending on betweenness. This was also validated by STRING, which resulted in a network of 36 nodes and 13 edges representing the interactions between the nodes, which could be physical, pathway, or co-expression interactions. The top five hub genes based on the node ranking scores were MMP7, REG1A, GUCA2A, UGT2B17, and DEFA6, with scores ranges from 0.6:0.4. In addition, STRING applies K-means clustering and provides three clusters of interacting genes, as shown in Figs. 4 and 5.

### Enrichment Analysis of CRC DEGS

**Fig. 6:**
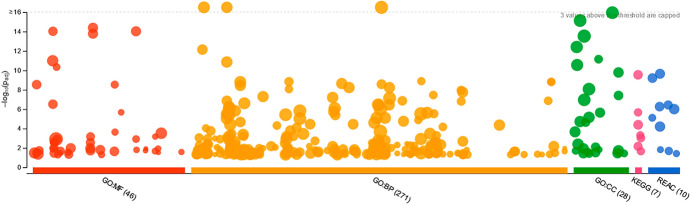
Enriched analysis of CRC DEGs

**Fig. 7:**
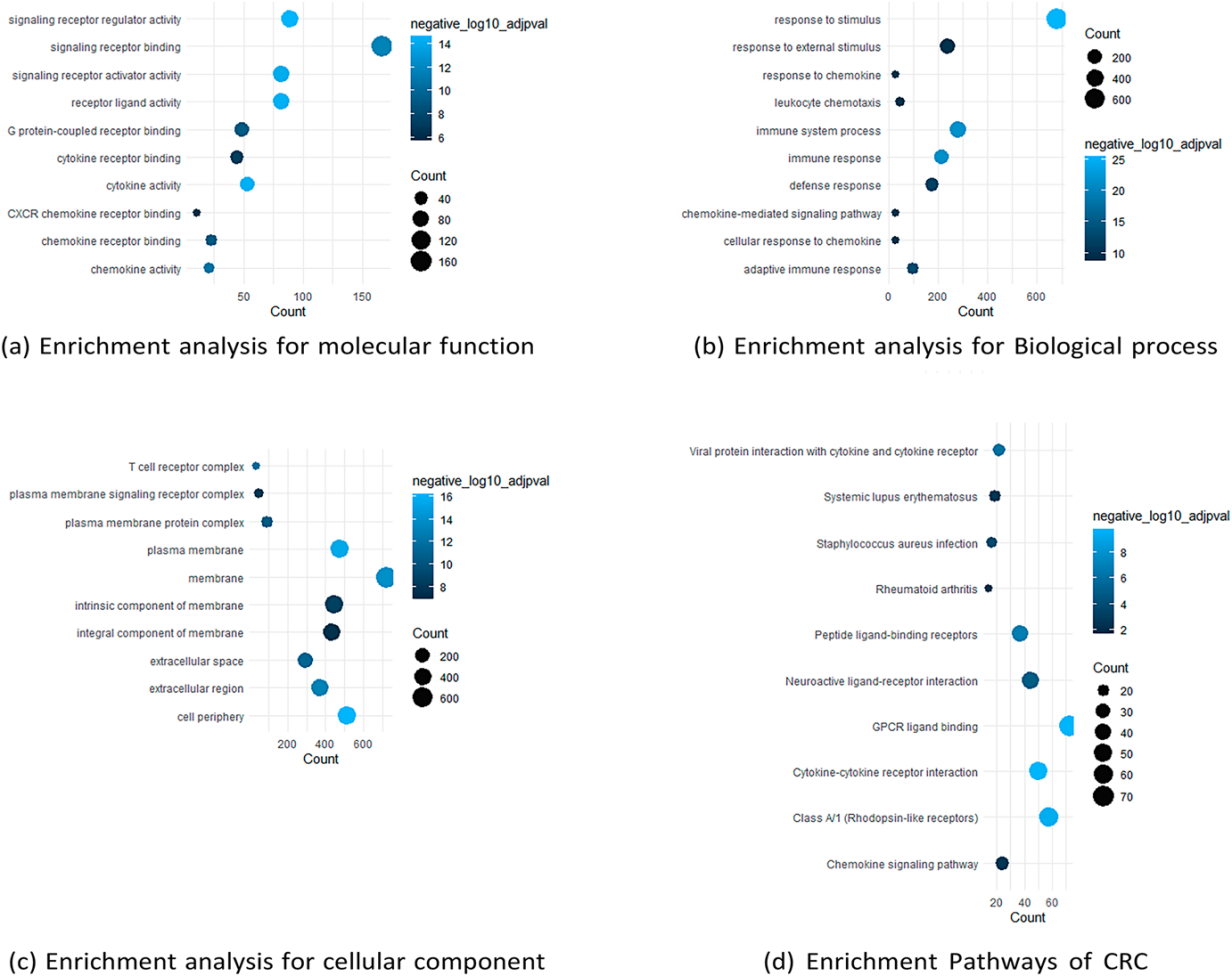
**a** Enrichment analysis for molecular function, **b** enrichment analysis for biological process, **c** enrichment analysis for cellular component and **d** enrichment pathways of CRC

Enrichment analysis of DEGs for CRC using g:Profiler software, the database identified 1573 out of 1641 genes and converted them to ID Entrez using GO Molecular function, GO Biological Process, GO Cellular Component, KEGG pathways, and Reactome Pathways, as shown in Fig. 6. Gene Ontology (GO) analysis indicated that alterations in the biological process (BP) of the DEGs were considerably enriched in the stimulus response, immune system process, and immune response. This study revealed that alterations in cell composition (CC) were predominantly concentrated in the cell periphery, with 512 genes exhibiting enrichment. Additionally, 476 genes were enriched in the plasma membrane, whereas 368 genes were enriched in the extracellular region. Differential expression analysis revealed that alterations in the MF of DEGs were predominantly enriched in three categories: signaling receptor regulator activity (89 genes), signaling receptor binding (166 genes), and cytokine activity (53 genes). KEGG pathway analysis revealed that DEGs were significantly enriched in cytokine–cytokine receptor interactions, chemokine signaling pathways, and neuroactive ligand–receptor interactions as shown in Fig. 7a–d.

The analysis showed that MMP7, AGT, ADORA3, VEGFD, and CCL2 are common genes in many biological processes, MFs, and pathways, in addition to those reported in the literature.

### Cytotoxicity Results

To determine the IC50 concentration for each treatment, cell viability was evaluated using the SRB assay and the absorbance was measured at 540 nm using a microplate reader (BMG LABTECH®- FLUOstar Omega model (Ortenberg, Germany) (Skehan et al. 1990; Allam et al. 2018).

**Fig. 8:**
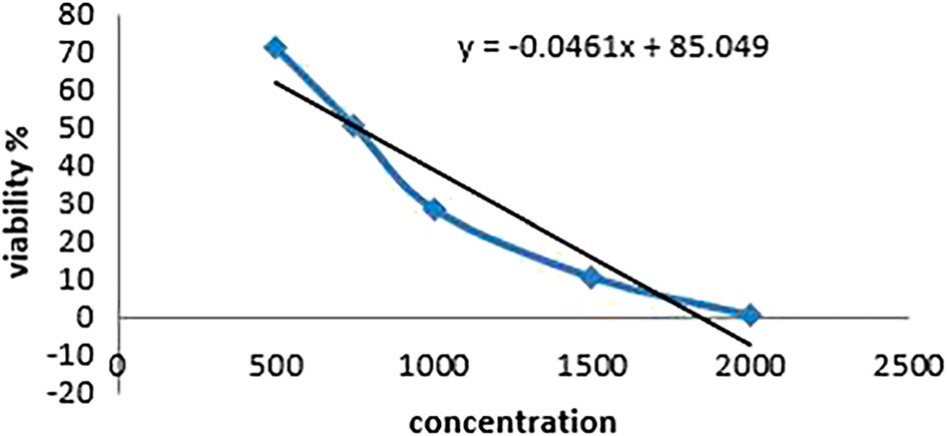
Concentration against cell viability in extract group

**Fig. 9:**
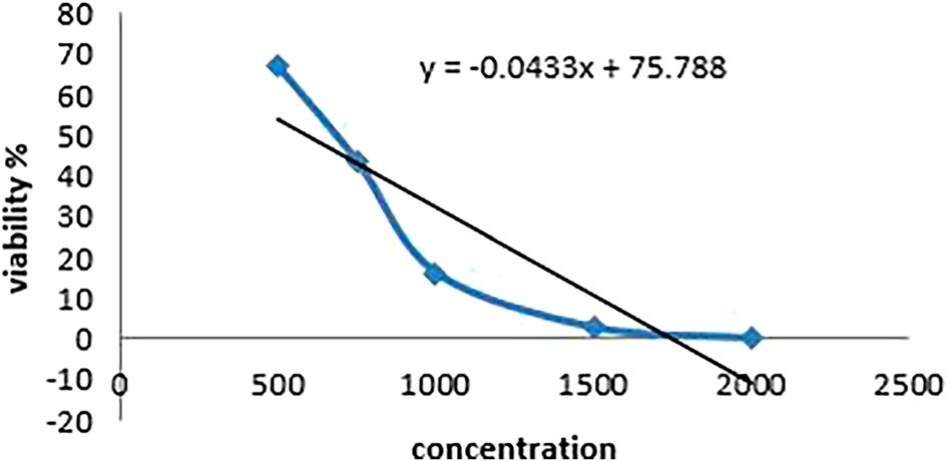
Concentration against cell viability in mixture group

**Fig. 10:**
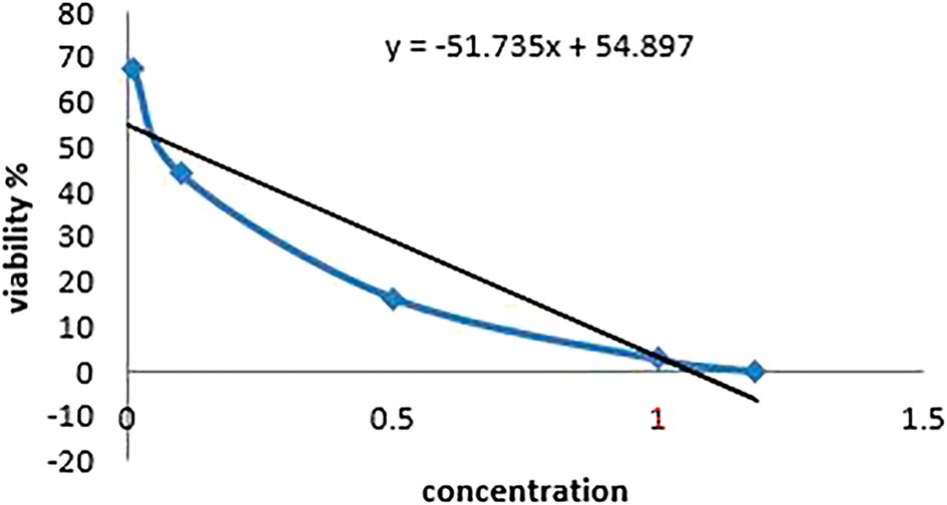
Concentration against cell viability in 5- FU group

The SRB test results in the following IC50 values for each treatment group are shown in Tables 4, 5 and 6 and Figs. 8, 9 and 10.

**Table 4:** Cell viability percentage with different WS extract concentrations used

Conc	Viability %
500	71.2347
750	50.1618
1000	28.2695
1500	10.2442
2000	0.18787
IC50	724

**Table 5:** Cell viability percentage with different concentrations between extract and 5-FU as a mixture

Conc	Viability %
500 + 0.01	67.2897
750 + 0.1	43.8603
1000 + 0.5	16.2527
1500 + 1	2.70378
2000 + 1.18	0.04148
IC50	595.69

**Table 6:** Cell viability percentage with different concentrations of 5-FU as chemotherapy

Conc	Viability %
0.01	67.2897
0.1	43.8603
0.5	16.2527
1	2.70378
1.18	0.04148
IC50	0.1

### Real-Time PCR Results

**Fig. 11:**
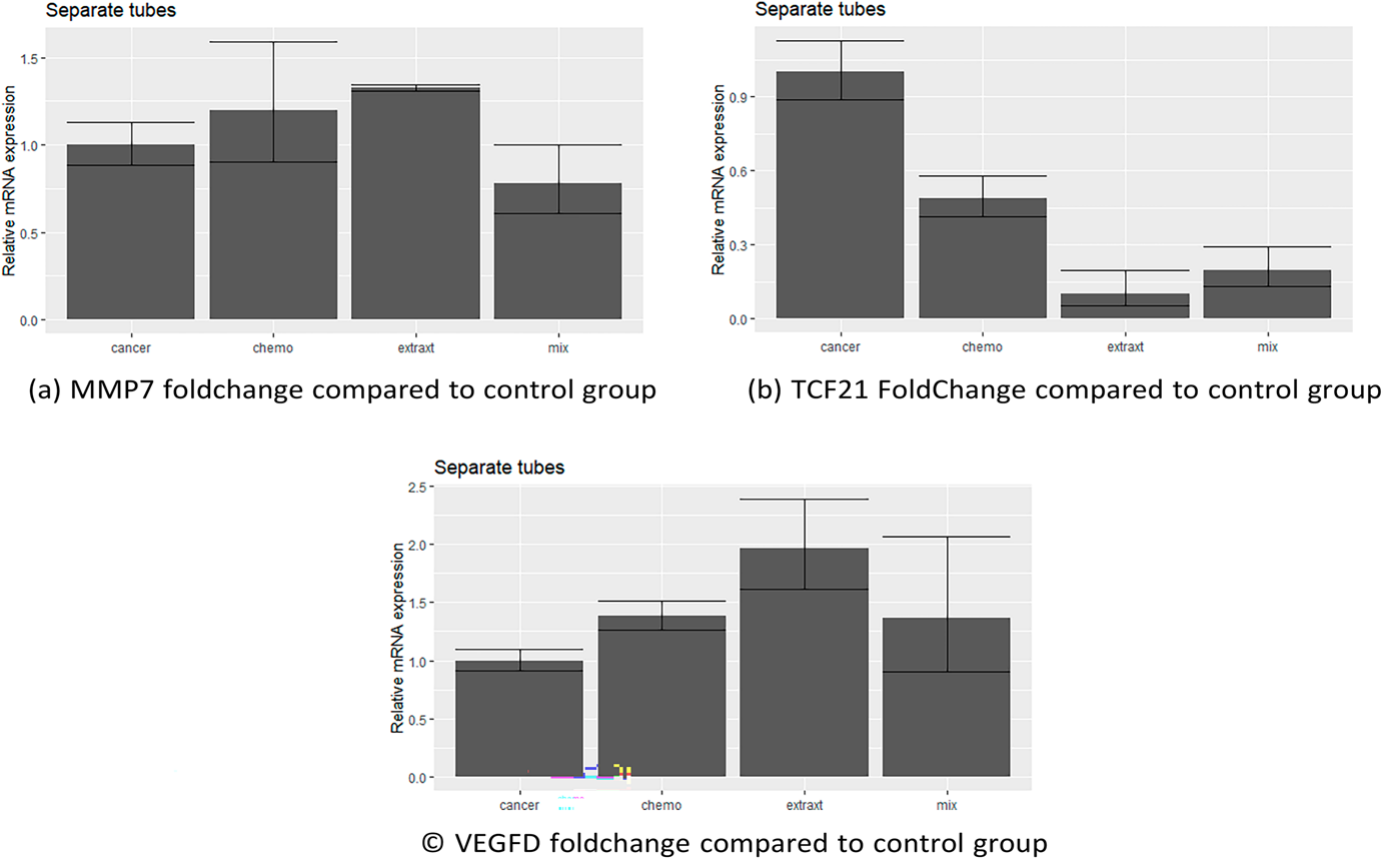
**a** MMP7 foldChange compared to control group, **b** TCF21 foldChange compared to control group and **c** VEGFD foldChange compared to control group

The resulting data were analyzed using the PCR analysis package in R, which uses $2^{ - \Lambda \Lambda C_{\text{t}} }$ method according to Hu et al. (2015), Schmittgen and Livak (2008) and is illustrated as bar graphs, as shown in Fig. 11a–c. Relative expression of MMP7 was 1.1, 1.3 and 0.7 in groups II, III, and IV, respectively. In contrast, the relative expression of TCF21 was 0.4, 0.09 and 0.1 for the three groups. In addition, the relative expression of the VEGFD gene was 1.4, 1.9 and 1.3 in the same three groups.

This also was proved by testing the significant difference depending on *p*-value using ANOVA test, Shapiro test and Tuckey test. For MMP7, The Shapiro test gives a *p*-value = 0.2818 which means that this data is normalized. Additionally after analyzing the data using both ANOVA and TukeyHSD tests, the results showed significance between group I and group IV with *p*-value = 0.0417 “*”. In case of VEGF-D gene, the results revealed that the WS extract group (III) gave the most up-regulation by 0.9 foldChange compared to control group (I), with significant *p*-value = 0.038 (**). However, the combination group (IV) was slightly over-expressed by 0.3 with no significant *p*-value.

Finally, for TCF-21 the results showed that the group III gave the most significant down-regulation by 0.91 foldChange compared to control group with significant *p*-value = 0.0000299 “**” by both ANOVA and Tuckey tests. In addition to Shapiro test *p*-value = 0.1197 which showed that this data is normalized. This can be concluded that there was significant difference between all groups.

### Molecular Docking Results

**Fig. 12:**
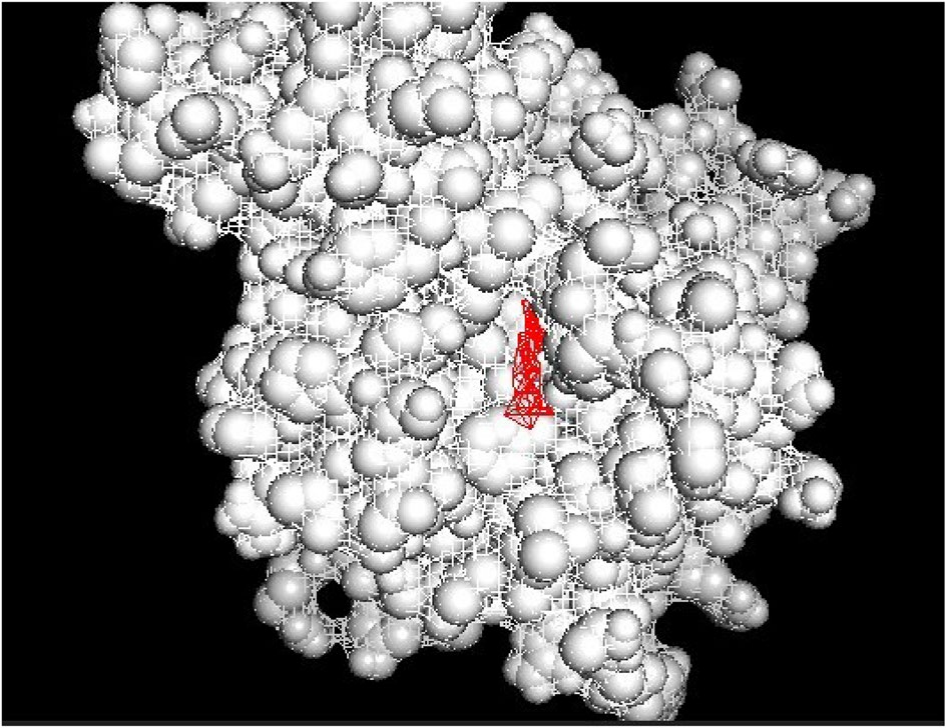
MMP7 active site docked with Withanolide A with MMP7

**Fig. 13:**
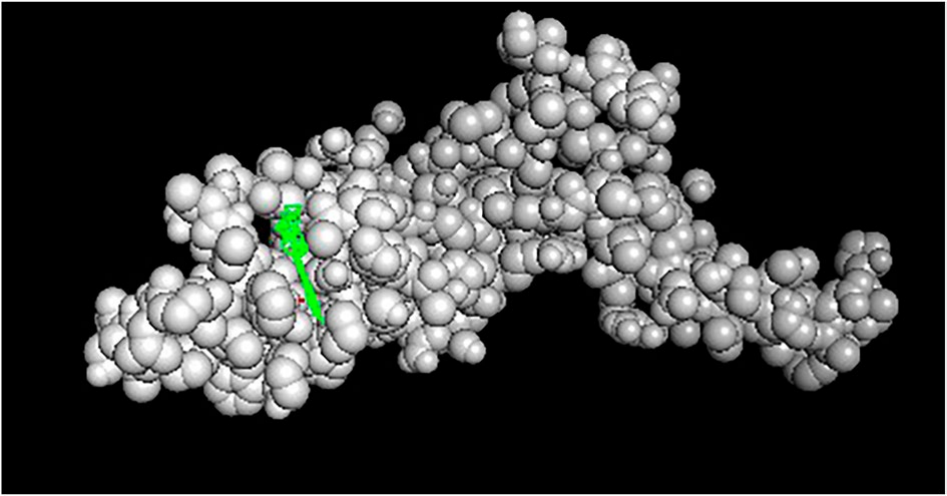
VEGFD active site docked with Withanolide A with VEGFD

**Fig. 14:**
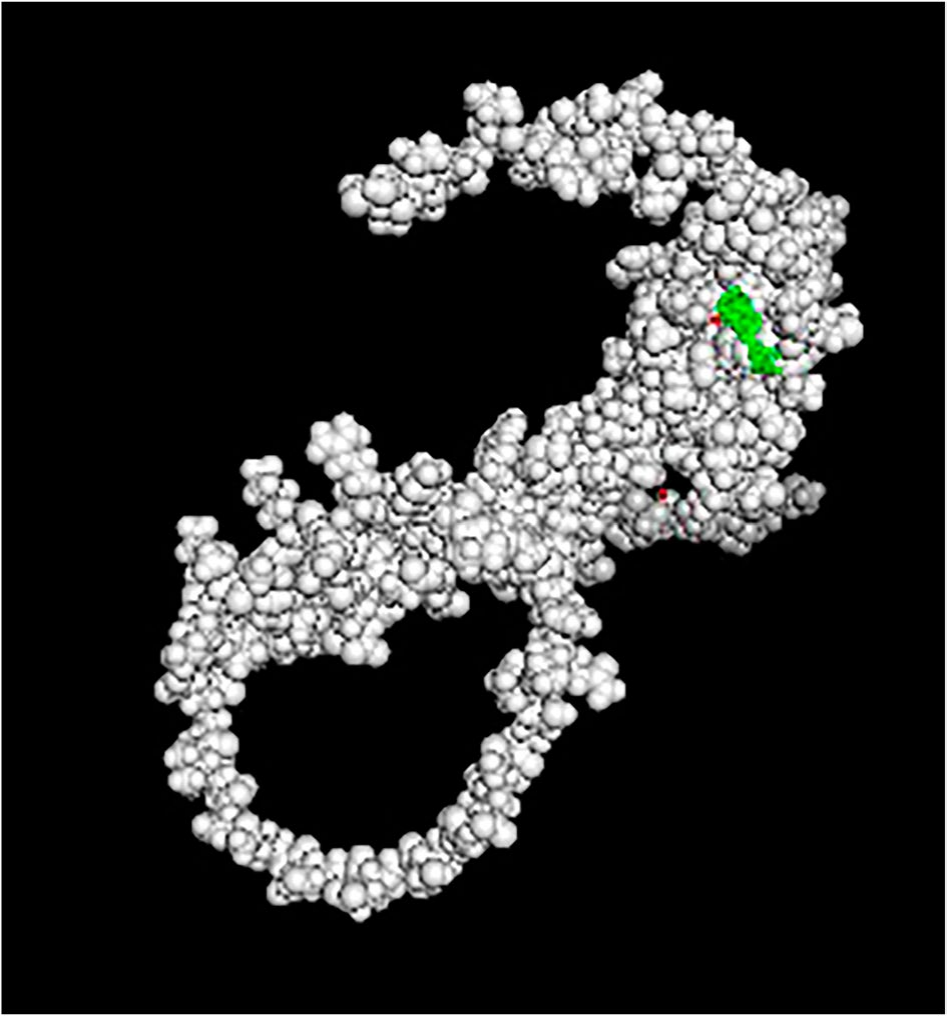
TCF21 active site docked with Withanolide A with TCF21

Withanolide A has a low binding affinity for MMP7 in the catalytic part of the protein (−12). Withaferin A has the lowest binding affinity with VEGFD and TCF21 in the catalytic part of the protein (−10.3 and −10.1, respectively) as shown in Figs. 12, 13 and 14 and Tables 7, 8 and 9 and these binding values revealed the strength of the interaction between those ligands and their target.

**Table 7:** Different binding affinities for ligands binding

Ligand name	Affinity
“Withanolide” A	−12.4
“Withaferin A”	−12.1
“Ginesing (Prosapogenin)”	−11.9
“Doxycycline”	−11
“Articulins (Ashweganda)”	−10
“Prinomastat”	−9.5
“EGRF Inhibitor”	−9.2
“Cabage Palm”	−8.8
“Apratastat”	−8.6
“Cipemastat”	−8.4
“Amobarbital”	−8
“Cyclizine”	−7.7
“Marimastat”	−7.1

**Table 8:** Different binding affinities for ligands binding

Ligand name	Affinity
“Withaferin A”	−10.3
“Withanolide A”	−10.2
“Ginesing (Prosapogenine”	−9.2
Ashweganda	−9
“Cabbage palm”	−6.8
“EGRF inhibitor”	−6.7
“Cyclizine”	−6.7
“Amobarbital”	−6.3
“5-fulurouracil”	−4.4

**Table 9:** Different binding affinities for ligands binding

Ligand Name	Affinity
“Withefirine A”	−10.1
“Ginesing (Prosapogenin)”	−9.8
“Withanolide A”	−9.4
“Ashweganda”	−9.1
“EGRF inhibitor”	−8.9
“Cyclizine”	−6.8
“Cabbage Palm”	−6.4
“5-fuloro uracil”	−5.5
“Amobarbital”	−5.4

## Discussion

CRC is a common gastrointestinal neoplasm that is distinguished by high rates of morbidity and mortality. Early diagnosis and corresponding intervention are considered the most effective approaches for increasing survival time and decreasing the mortality caused by CRC (Arhin et al. 2022; Khazaei et al. 2018). There is an unrelenting necessity for potential biomarkers discovery of this type of cancer to be identified, which will promote early detection and hence a higher treatment rate (Huo et al. 2018; Chen and Ke 2021). Several studies involving microarray analysis have documented the recognition of gene expression patterns in adenomas and cancers. To determine potential CRC DEGs, the gene expression profiles of GSE156451 were analyzed of 50 paired-end tumor samples and their adjacent normal tissue samples. We recognized 1641 DEGS (773 upregulated and 896 downregulated genes). To have a better understanding of the interactions between the resulted DEGS, we performed a functional enrichment analysis on GProfiler, The analysis showed that MMP7, AGT, ADORA3, VEGFD, and CCL2 are common genes in many biological process, MFs and pathways, which also reported in Chun et al. (2015); Ding et al. (2021); Ma et al. (2010). Then PPI-network was constructed using GeneMANIA-Cytoscape software and STRING database. About 20 hub genes were screened using CytoHubba MCC. Consequently, we chose three genes (MMP7, VEGFD and TCF-21) from the top 10 hub genes to be validated their relative expression on molecular level by q-PCR. Which resulted to be synchronized with the bio-informatics results. Additionally, it also necessary to know the ability to replace the traditional treatments (chemotherapy) with other treatments to avoid the side-effects of chemotherapy. We tested the extract of *Withania somnifera* plant in different concentrations by SRB cytotoxicity test, as it is recently known to exhibits anti-tumor capabilities. Finally, to validate the efficacy of W. *Withania somnifera*, molecular docking was performed by AutoDockvina to test the binding affinity of the target ligand (extract) in the catalytic part of the proteins (MMP7, VEGFD and TCF-21). Withanolides showed the lowest binding affinities, which indicates the effectiveness of them against the target proteins.

Based on functional enrichment analyses, the DEGs were significantly enriched in biological functions including immune response, immune system process, adaptive immune response, stimulus response, leukocyte migration, response to external biotic stimulus and response to stress A prior study identified immunological destruction, which leads to chronic inflammation, as a key cause of CRC; consequently, our transcriptome findings are consistent with earlier studies that found inflammation to be a major characteristic of the tumor microenvironment in CRC (Schottelius and Dinter 2006; Hammad et al. 2021).

Based on the construction of PPI network, MMP7, REG1A, GUCA2A, UGT2B17, and DEFA6, VEGFD, TCF-21, AGT, CCL2 and ADORA3 with a high degree of connectivity were identified as hub genes. Some of them were significantly downregulated in CRC tissues compared with normal tissues, while the others was significantly upregulated. The relative expression for three of them were validated by q-PCR as MMP-7 was upregulated in cancer cells (Chen and Ke 2021; Powell and Matrisian 1996). Furthermore, Fan et al. discovered that MMP7 was extremely important in the chemotherapeutic treatment of colon cancer (Peng et al. 2019). MMP was found to be significant in the epithelial-mesenchymal transition and invasion of colon carcinoma by Kobayashi et al. (Yamada et al. 2013). In addition, the association between MMP7 and invasive development of tumors as well as distant metastasis has been observed in colorectal tumors (CRC) (Sun et al. 2015). In this study, the treatment group IV cause significantly down- regulation by *p*-value 0.0417 compared to control group (I). This was also mentioned by Kyakulaga et al., which states that WS extract can target many cancer pathways as cytotoxicity, cell apoptosis, angiogenesis, inflammation, and immune modulation, they overlap in many forms of cancer (Kyakulaga et al. 2018).

Concerning VEGF-D, Vascular endothelial growth factors-D play crucial role as an angiogenic factor that control angiogensos process (Hanrahan et al. 2003). The angiogenic effects of VEGF-D have been demonstrated on endothelial cells in both in vitro and in vivo applications and its regulation is influenced by proinflammatory cytokines. Moreover, VEGF-C and VEGF-D are known to exert significant influence on the process of lymphangiogenesis (George et al. 2001). VEGFD was relatively up-regulated in the treated groups compared to the control group, which validated the in-silico results as the VEGFD showed to be down-regulated in cancer cells, as agreed with (Hanrahan et al. 2003; George et al. 2001). Moehler et al., recognized downregulation of VEGF-D after using cetuximab treatment (Moehler et al. 2008) and other study provides evidence that solid tumors can develop lymphatic vessels and suggests that VEGF family members play a crucial role in determining the pattern of metastasis by Stacker et al. (2001). However, our data didn’t come in line with other studies, there are many factors affecting VEGF-A and VEGF-D mRNA expression as age and VEGF-receptors (Hanrahan et al. 2003; Mazeda et al. 2020). The reduction of VEGF-D levels observed in polyps and carcinomas may facilitate the enhanced binding of VEGF-A and VEGF-C to the VEGF receptors (George et al. 2001). Moreover, all vascular endothelial growth factors A, B, and C showed low levels of expression in elderly patients, except for VEGF-D, which was slightly down-regulated in younger ages than old ages with no significant results (Mazeda et al. 2020; Ito et al. 2005), given that according to American Type Culture Collection (ATCC), our used HCT-116 cell line were collected from adult patients. In addition, Wang et al. said that the formation and development of tumors are influenced by inflammation, which operates through several mechanisms such as the release of inflammatory mediators, vascular abnormalities, and epigenetic changes that modify gene expression (Mazeda et al. 2020). This was proved by our previously mentioned enrichment analysis as VEGF-D was significantly enriched in many BP concerning the immune response and immune system process.

Finally, TCF-21 plays a crucial role an anti-angiogenic factor and recognized as a tumour suppressor in various types of cancers, as demonstrated by Arab et al. (2011). The up-regulation of TCF21 has been found to impede the motion of melanoma cells. Additionally, it has been observed to diminish the proliferation of cancer cells and the formation of colonies in lung and cervical cancers. In contrast to literature, the current work showed significant downregulation of TCF-21 among the treatment groups compared to control group. This was preceded by in-silico studies with the same conclusion that TCF-21 is a gene that is up-regulated in cancer cells. This may be explained by Dai et al. (2016), where they stated that some treatments activate the hyper-methylation promoter which cause dysregulation of TCF-21. Dia et al., has shown in their work that the di-activation of methylation promotor will regulate the expression of TCF-21 to do its anti-angiogenesis role normally. This also was proved by Jones (2002), the tumor suppressor genes are largely silenced as a result of alterations in DNA methylation. From other perspective, TCF-21 may be dysregulated by the action of VEGF group, it was mentioned by Chen et al. in 2019 that VEGF could boost cancer cells’ production of miR-205 which was found to inhibit the action of TCF-21 (Chen et al. 2019).

In conclusion, this analysis pipeline is robust and accurate in identifying the most notable CRC DEGs consistent with the literature. The identified genes can be used as potential biomarkers for CRC diagnosis and treatment as mentioned before early recognition of precancerous lesions in the colorectal is significantly important to enhance the survival rate and also help in timely treatment. Additionally, our research may offer novel approaches for analyzing the most significant biological functions in CRC focusing on the immune response. Furthermore, those biomarkers can be used later on studies with an increased sample size for survival analysis of CRC and cross-analysis using different types of Geo datasets. Nevertheless, this study does possess several drawbacks. First, there is further trials needed to test the regulation of the methylation promoters and also overcome many clinical challenges to clinically translate the in vitro results as the clinical information on the databases is not enough due to the absence of comprehensive grade data pertaining to patients diagnosed with CRC, as well as the limited availability of follow-up information subsequent to surgical interventions, presents a significant gap in current academic research. In addition, additional investigation needed to explore the therapeutic potential of biomarkers that have been found which will be also the focal point of our subsequent research endeavors. Finally, it is anticipated that this study’s results will contribute to the identification of novel diagnostic and prognostic biomarkers, as well as potential therapeutic targets, for CRC.

### Supplementary Information

Below is the link to the electronic supplementary material.

Supplementary file1 (PDF 342 KB)

## Data Availability

The data are available under accession number in NCBI, Gene Expression Omnibus (GEO): GSE156451.
