# Supplementary material for: Identification of Differentially Expressed Genes in Human Colorectal Cancer Using RNASeq Data Validated on the Molecular Level with Real-Time PCR
Source: Biochem Genet. 2023 Dec 14;62(4):3260–84. doi: 10.1007/s10528-023-10593-5 (PMC11289010; doi:10.1007/s10528-023-10593-5)

In-vitro Experiment details:

SRB cytotoxicity test: the concentration used for %-FU and WS- extract:

Extract	Raw data			Blank Corrected Data			Viability %				
Conc	1	2	3	1	2	3	1	2	3	Mean	STD
**c**	6.241	6.311	6.889	6.148	6.218	6.796	100	100	100	100	0
**500**	4.721	4.589	4.619	4.628	4.496	4.526	72.4559	70.3893	70.859	71.2347	0.88452856
**750**	3.296	3.332	3.263	3.203	3.239	3.17	50.1461	50.7097	49.6295	50.1618	0.44115446
**1000**	1.871	1.886	1.939	1.778	1.793	1.846	27.8363	28.0712	28.9009	28.2695	0.4566851
**1500**	0.785	0.719	0.738	0.692	0.626	0.645	10.8339	9.80065	10.0981	10.2442	0.43431058
**2000**	0.101	0.094	0.12	0.008	0.001	0.027	0.12525	0.01566	0.42271	0.18787	0.17197847
**Blank**	0.093	0.093	0.093	Blank Average		**0.093**	**Control average**		**6.38733**		

Extract +	Raw data			Blank Corrected Data			Viability %				
Conc	1	2	3	1	2	3	1	2	3	Mean	STD
**c**	6.74	6.775	6.784	6.64333	6.67833	6.68733	100	100	100	100	0
**500+0.01**	4.756	4.64	4.358	4.65933	4.54333	4.26133	69.8586	68.1193	63.8912	67.2897	2.50578285
**750+0.1**	3.17	2.961	2.935	3.07333	2.86433	2.83833	46.0793	42.9457	42.5558	43.8603	1.57712108
**1000+0.5**	1.237	1.107	1.198	1.14033	1.01033	1.10133	17.0973	15.1482	16.5126	16.2527	0.81666983
**1500+1**	0.321	0.267	0.243	0.22433	0.17033	0.14633	3.36349	2.55385	2.19401	2.70378	0.48906511
**2000+1.18**	0.1	0.0993	0.099	0.00333	0.00263	0.00233	0.04998	0.03948	0.03498	0.04148	0.00628208
**Blank**	0.096	0.097	0.097	Blank Average		**0.09667**	**Control average**		**6.66967**		

qPCR detailed methodology:

RNA extraction and cDNA synthesis

The total RNA was extracted and purified from the provided cell line pellets utiliz- ing the Qiagen RNeasy Mini kit (Cat No.74104) following the manufacturer’s instructions. The RNA samples were quantified and their quality assessed using a Nanodrop spec- trophotometer at A230, A260, and A280. First-strand cDNA synthesis was performed on RNA samples obtained from the four groups using the RevretAid first-strand cDNA synthesis kit (Cat K1622, Thermoscientific^{TM}) according to the manufacturer’s instruc- tions. The cDNA synthesis was carried out using a Bio-Rad TM 100 Thermal cycler. The

following components were added in the same order to a sterile PCR-tube:

	cDNA reaction component	Volume/Amount
1	RNA	3 ug
2	Oligo dT (100 pmol)	1 ul
3	ddH_2O	up to 12 ul

Table: cDNA synthesis componenets

The tube were incubated at 65 °C for 5 minutes, followed by immediate coolingin ice for few minutes. the following components were immediately added to each tube while in ice:

	cDNA reaction component	Volume/Amount
1	5X Enzyme Buffer	4ug
2	Riboblock RNAse inhibtor (20U/μl)	1 ul
3	dNTPs (10mM)	2 ul
4	RevertAid RT-enzyme	1 ul

Table: cDNA synthesis componenets

Real-time PCR to test gene expression

The cDNA was thereafter amplified with the TB green Permix EXTTaq PCR Master Kit in a 48-well plate using the Stratagene Mx3005P, Agilent Technologies as follows: 30 sec. at 95 °C for enzyme activation followed by 45 cycles of 5 seconds at 95 °C, 30 seconds at 60 °C and 30 second at 72 °C for the amplification step. We used 0.25 uM of both primers specific for each target gene. The studied genes primers' sequences were as follows:

Gene	Primer Sequence
MMP7 (F)	TCGGAGGAGATGCTCACTTCGA
MMP7 (R)	GGATCAGAGGAATGTCCCATACC
TCF21 (F)	ATCCTGGCTAACGACAAATACG
TCF21 (R)	CACTTCTTTCAGGTCACTCTCG

VEGFD (F)	GACTGGAAGCTGTGGAGATGCA
VEGFD (R)	GGCTGCACTGAGTTCTTTGCCA

Table: Gene Primer Sequence

The total volume of the reaction was completed with $ddH2_{O}$ to be 20 μl. The relative gene expression foldChange was calculated using PCR analysis R package de- pending on the equation of $2^{-\Delta\Delta ct}$. The following components were added in the sameorder:

qPCR reaction component	Volume
TB green Permix EX Taq™	5 μl
Rox dye	0.2 μl
Forward Primer (5pmol)	0.25 μl
Reverse Primer (5pmol)	0.25 μl
cDNA	1.3 μl

The 2D pictures of docking:

MMP7:

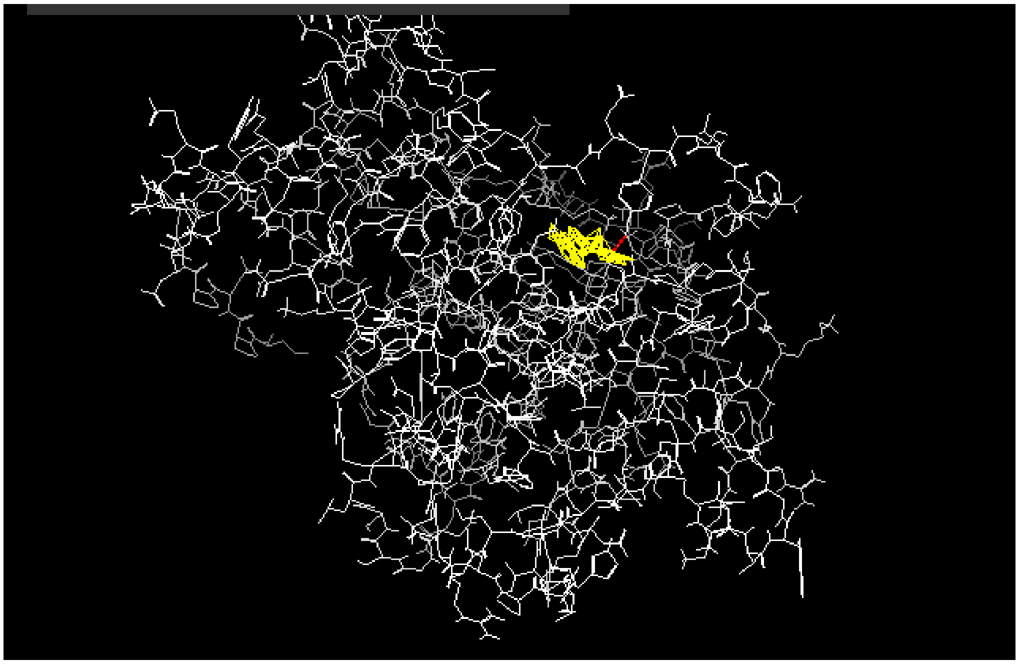

VEGFD:

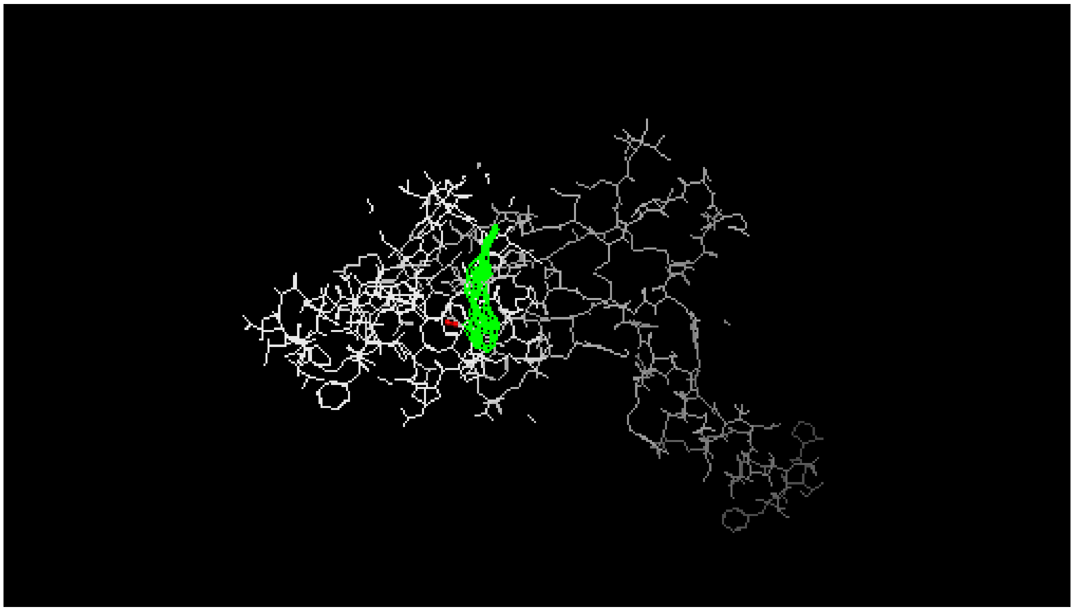

TCF21:

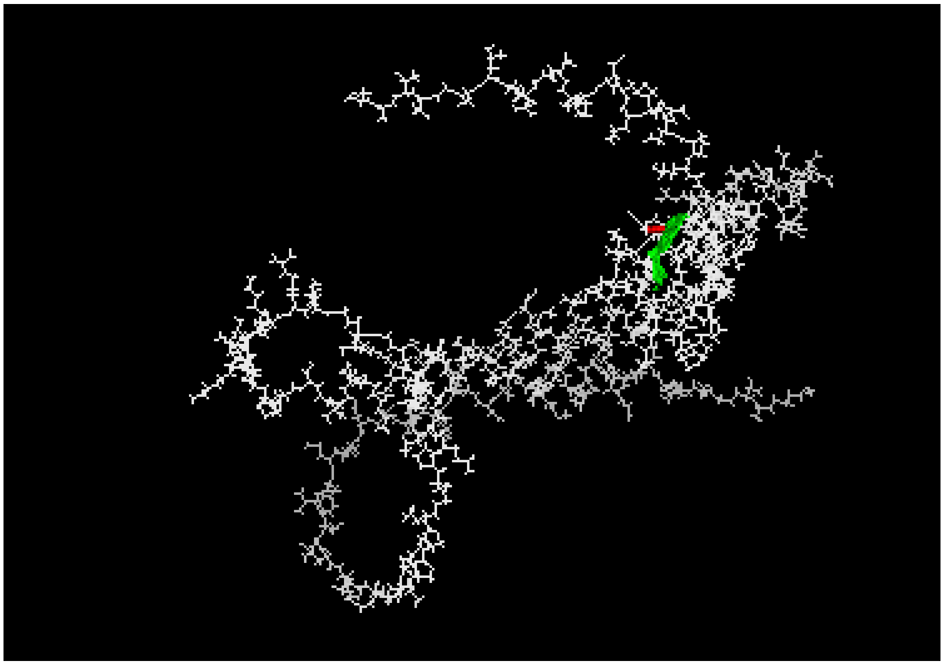

Supplement: Supplementary file 1 — Supplementary file1 (PDF 342 KB) [file 10528_2023_10593_MOESM1_ESM.pdf]
